# Phenotypic Variability in Novel Doublecortin Gene Variants Associated with Subcortical Band Heterotopia

**DOI:** 10.3390/ijms25105505

**Published:** 2024-05-18

**Authors:** Radha Procopio, Francesco Fortunato, Monica Gagliardi, Mariagrazia Talarico, Ilaria Sammarra, Maria Chiara Sarubbi, Donatella Malanga, Grazia Annesi, Antonio Gambardella

**Affiliations:** 1 Department of Medical and Surgical Sciences, Neuroscience Research Center, Magna Graecia University, 88100 Catanzaro, Italy; radha.procopio@unicz.it (R.P.); monica.gagliardi@unicz.it (M.G.); 2 Department of Medical and Surgical Sciences, Institute of Neurology, Magna Graecia University, 88100 Catanzaro, Italy; francescofortunato@unicz.it (F.F.); mary.talarico21@gmail.com (M.T.); ilaria.sammarra@unicz.it (I.S.); 3 Laboratory of Molecular Oncology, Department of Experimental and Clinical Medicine, Magna Graecia University, 88100 Catanzaro, Italy; mariachiarasarubbi93@gmail.com (M.C.S.); malanga@unicz.it (D.M.); 4 Interdepartmental Center of Services (CIS), Magna Graecia University, 88100 Catanzaro, Italy; 5 Institute for Biomedical Research and Innovation, National Research Council, 87036 Cosenza, Italy

**Keywords:** *DCX*, doublecortin, Subcortical Band Heterotopia (SBH)

## Abstract

Doublecortin, encoded by the *DCX* gene, plays a crucial role in the neuronal migration process during brain development. Pathogenic variants of the *DCX* gene are the major causes of the “lissencephaly (LIS) spectrum”, which comprehends a milder phenotype like Subcortical Band Heterotopia (SBH) in heterozygous female subjects. We performed targeted sequencing in three unrelated female cases with SBH. We identified three DCX-related variants: a novel missense (c.601A>G: p.Lys201Glu), a novel nonsense (c.210C>G: p.Tyr70*), and a previously identified nonsense (c.907C>T: p.Arg303*) variant. The novel c.601A>G: p.Lys201Glu variant shows a mother–daughter transmission pattern across four generations. The proband exhibits focal epilepsy and achieved seizure freedom with a combination of oxcarbazepine and levetiracetam. All other affected members have no history of epileptic seizures. Brain MRIs of the affected members shows predominant fronto-central SBH with mixed pachygyria on the overlying cortex. The two nonsense variants were identified in two unrelated probands with SBH, severe drug-resistant epilepsy and intellectual disability. These novel DCX variants further expand the genotypic–phenotypic correlations of lissencephaly spectrum disorders. Our documented phenotypic descriptions of three unrelated families provide valuable insights and stimulate further discussions on DCX-SBH cases.

## 1. Introduction

The *DCX* gene, located on Xq22.3, encodes for a protein called “doublecortin”, primarily implicated in the neuronal migration process during brain development [1,2,3,4]. Doublecortin is expressed in differentiating neurons and plays a pivotal role in microtubule organization [1,2,3,4]. Pathogenic DCX variants lead to a defective neuronal migration, resulting in disorganized cortical layers [1,2,3,4]. The majority of these variants are concentrated within two evolutionarily conserved domains: the N-terminal domain (N-DC, amino acids 42–150) and the C-terminal domain (C-DC, amino acids 171–275). Both domains are essential for effectively regulating microtubule function during neuronal migration. N-DC specifically binds to assembled microtubules, whereas C-DC binds to both microtubules and unpolymerized tubulin. A recent study indicated that missense variants in the N-DC tend to induce more severe abnormalities compared to the variants in the C-DC [5].

Pathogenic variants of the *DCX* gene, together with *LIS1* and tubulin genes, are the major causes of the “lissencephaly (LIS) spectrum”, a group of malformations of cortical Development (MCD) with epileptic seizures and a various range of intellectual disability caused by impaired neuronal migration in the early stages of embryonic development [6,7,8].

Due to its location on the X chromosome, the severity of the LIS spectrum is predominantly influenced by gender [1,2,3,4]. In hemizygous males, where there is only one copy of the X chromosome, all neural cells carry the mutation. This leads to more severe MCD such as lissencephaly. On the other hand, heterozygous female subjects usually exhibit a milder phenotype, such as Subcortical Band Heterotopia (SBH) [9].

SBH is characterized by bands of grey matter that are interposed in the white matter between the cortex and the lateral ventricles with a normal or simplified gyral pattern of the overlying cortex [6,7]. Currently, according to Di Donato’s latest classification, the LIS spectrum is mainly classified based on the severity and gradient of gyral malformation, the thickness of the heterotopic band, the pattern of the overlying cortex and the presence of associated malformations [10,11].

Furthermore, the severity of neurological impairment, epileptic seizures and intellectual disability were found to be correlated with the degree of gyral pattern of cortical malformation as well as the thickness of the heterotopic band detected by brain magnetic resonance imaging (MRI) [3]. The International League Against Epilepsy (ILAE) Neuroimaging Task Force has recently addressed the challenges of diagnosing and managing SBH cases, emphasizing the value of appropriate high-resolution MRI protocols [12].

Several genetic variations, including missense, nonsense, frameshift, and deletions, were documented in both familial and sporadic cases, either as inherited mutations or de novo mutations. Inherited mutations, often missense, tend to result in less severe phenotypes compared to nonsense mutations, which lead to protein truncations [13,14]. Numerous studies propose that skewed X inactivation or low-level mosaicism may also contribute to the heterogeneity of phenotypes within families, especially among asymptomatic carriers. Specifically, low-level mosaicism was identified as an under-recognized factor in apparent de novo germline mutations [15].

In this context, we conducted targeted sequencing within our epilepsy cohort to identify DCX causative variants. Our investigation centered on establishing genotypic–phenotypic correlations within three unrelated female cases displaying SBH.

## 2. Results

### 2.1. Electroclinical and Neuroimaging Data

#### 2.1.1. Family 1

**Figure 1:**
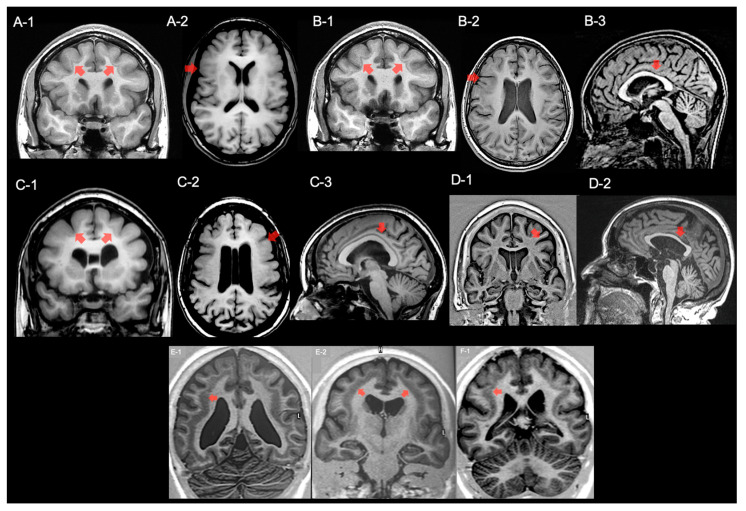
Brain MRI changes of Family 1, 2 and 3. Coronal (**A-1**) and axial (**A-2**) T1-weighted images of the proband (F1-III-1) of Family 1 showed a fronto-central SBH with mixed pachygyria (red arrows). Coronal (**B-1**), axial (**B-2**) and sagittal (**B-3**) T1 images of the F1-III-2 also showed a fronto-central SBH with mixed pachygyria together with a hypoplastic aspect of corpus callosum and cavum vergae (red arrows). Brain MRI (**C-1**–**C-3**) of the (F1-II-1) showed the same findings of the proband. Notice the dysmorphic aspect of corpus callosum (**C-3**, red arrow). MRI findings of the F1-I-1 with fronto-central SBH with mixed pachygyria and a dysmorphic aspect of corpus callosum as well (**D-1**,**D-2**). Coronal T1-weighted images of the proband (F2-II-1) of Family 2 show a bi-hemispheric fronto-parietal pachygyria with a thick SBH (**E-1**,**E-2**). Also notice the thick band of SBH of the proband (F3-II-1) of Family 3 (**F-1**, red arrow).

**Figure 2:**
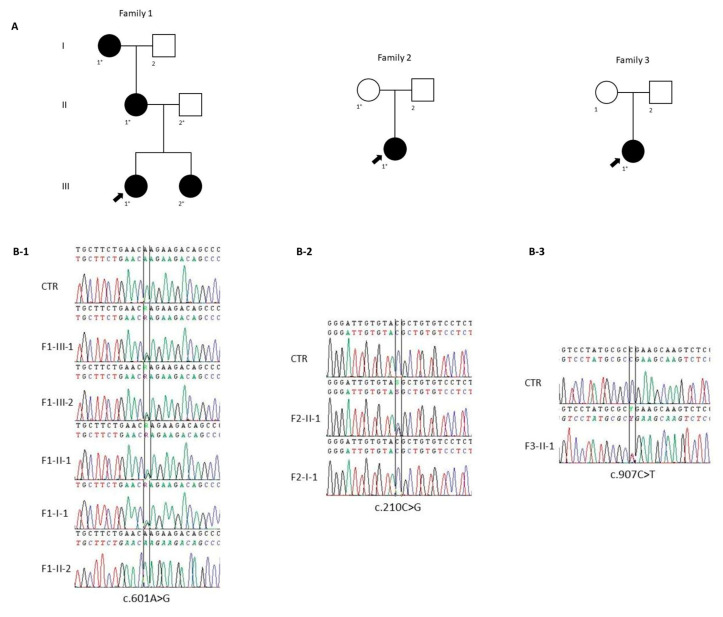
Pedigree of the families recruited and DCX sequence variants. (**A**) The fully filled symbols represent the affected individuals; unfilled symbols, unaffected; arrow, proband; plus, individuals undergoing genetic analysis. (**B-1**) Electropherogram shows the wildtype sequence (at the top), the c.601A>G variant in proband F1-III-1 and her affected family members (sister F1-III-2, mother F1-II-1, and grandmother F1-I-1) (middle), and the wildtype sequence in her unaffected father F1-II-2 (at the bottom). (**B-2**) The wildtype sequence (at the top), the c.210C>G variant in heterozygous proband F2-II-1 (middle), and the wildtype sequence in her unaffected mother F2-I-1 (at the bottom). (**B-3**) The wildtype sequence (above) and c.907C>T variant in heterozygous proband F3-II-1 (below).

The proband (F1-III-1) is a 35-year-old woman with focal epilepsy since the age of 9 years. She experienced two types of seizures: focal aware seizures characterized by an experience of “choking”, flushing, staring and right-eye deviation occurring approximately 1–2 times per month; focal to bilateral tonic–clonic seizures occurring approximately once every year. Seizure freedom was achieved with a combination of oxcarbazepine 1500 mg/d and levetiracetam 2000 mg/d. Her intelligence quotient (IQ) was 64 and her Mini-Mental State Examination (MMSE) was 26/30. The neurological examination, as well as the physical exam, were unremarkable. Interictal EEG demonstrated diffuse epileptiform discharges with left fronto-central-temporal predominance. A brain 3-T MRI showed bilateral predominant fronto-central SBH with mixed pachygyria on the overlying cortex (Figure 1(A-1,A-2)).

The proband’s sister (F1-III-2) is a 26-year-old woman with no previous history of seizures. She had mild intellectual disability (IQ 60) and a psychiatric disorder. Her neurological and physical examinations were otherwise normal with unremarkable standard EEG. A brain 3-T MRI showed the same findings as the proband plus a hypoplastic aspect of corpus callosum and cavum vergae (Figure 1(B-1–B-3)).

The proband’s mother (F1-II-1) is a 59-year-old woman with chronic headaches. She never had seizures. Her neuropsychological profile and IQ were normal. Her neurological examination was unremarkable and no other abnormalities were found on physical examination. A brain MRI showed fronto-central SBH-pachygyria, and a dysmorphic aspect of the corpus callosum and cavum vergae (Figure 1(C-1–C-3)).

The proband’s grandmother (F1-I-1) is a 78-year-old woman with no seizures or other neurological diseases. Neurological examination was normal and no other abnormalities were found on physical examination. Her neuropsychological profile and IQ were normal. The brain MRI showed fronto-central SBH-pachygyria and a hypoplastic aspect of the corpus callosum and cavum vergae (Figure 1(D-1,D-2)).

#### 2.1.2. Family 2

The proband (F2-II-1) is a 39-year-old woman with severe drug-resistant epileptic encephalopathy and intellectual disability (IQ 55), with her first seizure at the age of 3 years. She experienced atypical absences; focal seizures with staring, behavioral arrest, and tonic head deviation and focal to bilateral tonic–clonic seizures. Daily seizures persisted despite polytherapy with valproate 1500 mg/d, clobazam 30 mg/d, and lamotrigine 100 mg/d. Her neurological examination was otherwise normal. An EEG showed diffuse as well as focal epileptiform discharges involving temporo-occipital regions. A brain 3-T MRI revealed bi-hemispheric fronto-parietal pachygyria with thick SBH (Figure 1(E-1,E-2)). Her mother had an unremarkable clinical history with normal EEG, as well as a brain MRI. Her father declined genetic and neuroimaging investigations, and other family members reported no significant clinical signs.

#### 2.1.3. Family 3

The proband (F3-II-1) was a 40-year-old woman affected by severe focal drug-resistant encephalopathy with intellectual disability and a psychiatric disorder. Her seizures started at 10 years old with several types of seizures: focal unaware seizures characterized by fear, staring, tonic eye and head deviation; focal to bilateral tonic–clonic seizures; drop seizures with abrupt falls. Daily seizures persisted despite polytherapy with lamotrigine 600 mg/d, lacosamide 200 mg/d, and rufinamide 400 mg/d. An EEG showed diffuse as well as focal interictal discharges with right fronto-centro-temporal predominance. A brain 3-T MRI demonstrated bilateral frontal pachygyria and SBH with anterior predominance (Figure 1(F-1)).

### 2.2. Genetic Data

Next-generation sequencing was performed using the Ion Torrent platform with an AmpliSeq custom panel, designed to sequence the coding regions of 39 genes (Supplementary Table S1), previously reported to contain disease-causing mutations in cases with lissencephaly and epilepsy.

Single-nucleotide variants were predicted using the algorithms described in cases and methods section, while all synonymous, 5′UTR and 3′UTR variants were not considered in this study. Three variants in the *DCX* gene were identified in three unrelated cases: c.601A>G (p.Lys201Glu), c.210C>G (p.Tyr70*), and c.907C>T (p.Arg303*). Sanger sequencing analysis confirmed the presence of these variants.

The c.601A>G (p.Lys201Glu), present in exon 3, was found in a heterozygous state (Figure 2(B-1)) in F1-III-1. Segregation analysis revealed the presence of the same variant in F1-III-2, F1-II-1 and F1-I-1, but not in her father. This variant was not found in any population databases examined, nor among the 250 healthy subjects. It causes a missense lysine to glutamic acid substitution in position 201 of the protein. To evaluate the evolutionary conservation of lysine 201, we performed a protein alignment which revealed strong conservation from *C. elegans* to humans. Mutation Taster, PolyPhen2, REVEL and AlphaMissense predicted a pathogenic role of the variant. Thus, according to American College of Medical Genetics and Genomics (ACMG) guidelines we classified the variant as “likely pathogenic”.

The c.210C>G variant (rs587783532), present in exon 2, was found in a heterozygous state (Figure 2(B-2)) in F2-II-1, while it was absent in her mother (F2-I-1). This variant is not present in the gnomAD database and was not found in our cohort of 250 controls. It leads to premature termination of translation and it was predicted as a pathogenic variant by Mutation Taster. Thus, according to ACMG guidelines, we classified the variant as “pathogenic”.

The c.907C>T variant (rs587783592), present in exon 5, was found in a heterozygous state (Figure 2(B-3)) in the proband of Family 3 (F3-II-1). This variant was previously reported as a pathogenic variant [16]. It is not present in the gnomAD database and in our cohort of 250 healthy subjects. It led to a change from arginine to a stop codon at amino acid number 303, and according to ACMG guidelines, we classified the variant as “pathogenic”.

All the in silico analyses and applied criteria are summarized in Table 1.

We did not detect mutations in other genes, and we excluded deletions and duplications in the *DCX* and *LIS1* genes by MLPA in all cases.

## 3. Discussion

More than 150 different variants of the DCX gene were reported, with a prevalence of missense variants (64.7%), followed by small deletions (8.5%), gross deletions (7.8%), nonsense variants (7.2%), small insertions (4.6%), splicing variants (4.6%), gross insertions (1.3%), small indel (0.7%), and complex rearrangement (0.7%) [14]. Missense variants are typically inherited, clustered in the evolutionarily conserved domains (N-DC and C-DC) and tend to cause less severe phenotypes than nonsense variants, which are often found in sporadic cases [13].

In our investigation, we conducted a comprehensive screening of all coding regions of the *DCX* gene through targeted panel sequencing. Our findings revealed three distinct variants in three unrelated probands. In Families 1 and 2, we identified two novel pathogenic variants within the *DCX* gene. Additionally, in Family 3, we observed a unique phenotype associated with a previously documented variant.

The novel c.601A>G (p.Lys201Glu) variant identified in Family 1 segregates in a four-generation pedigree with mother–daughter DCX transmission. The variant impacts the C-DC, a critical domain for doublecortin function, as it binds microtubules and unpolymerized tubulin [5]. Specifically, lysine 201, an amino acid with a positively charged side chain, exhibits a high degree of conservation across various species, suggesting its pivotal role in the functionality of the protein. The pathogenic conversion of lysine 201 to glutamic acid, with a negatively charged side chain, could alter all the interactions. Moreover, previous studies have demonstrated that variants found in the C-DC, including p.Lys202Met and p.Thr203Ale, both near our p.Lys201Glu variant, significantly decreased microtubule binding ability, affecting polymerization in cells [5]. Multiple in silico tools also support the highly damaging effect of the c.601A>G (p.Lys201Glu) variant. The novel AlphaMissense tool further predicted a highly deleterious impact with a pathogenicity score of 0.9989, supporting the variant’s significance [17]. Consequently, we posit that this novel variant comprehensively accounts for the observed phenotype in affected females from Family 1. All the affected females share a mild phenotype compared to the more pronounced brain MRI features. Specifically, the proband exhibits well-controlled focal epilepsy, while all other affected members have no history of epileptic seizures. The proband’s sister presents mild intellectual disability along with a psychiatric disorder, the proband’s mother experiences only chronic headaches, and the grandmother has no history of neurological disease. Notwithstanding these mild clinical features, all affected members in Family 1 display SBH with mixed pachygyria on the overlying cortex. Additionally, three individuals exhibit dysmorphic or hypoplastic aspects of the corpus callosum. MRI abnormalities of the corpus callosum, such as complete agenesis, were documented in the LIS-SBH spectrum and are also incorporated in the latest imaging criteria for LIS-SBH [10]. However, unlike our cases, malformations of the corpus callosum were frequently accompanied by hypoplastic basal ganglia abnormalities as well as associated with the most severe spectrum of lissencephaly [10].

The novel truncating variant c.210C>G (p.Tyr70*) in Family 2 was exclusively present in the proband. This variant affected the N-DC and likely significantly impaired doublecortin’s ability to bind microtubules. Sanger sequencing of her unaffected mother did not reveal the variant, suggesting a potential de novo transmission. Although we did not investigate other possibilities, such as a skewed X inactivation as well as low-level mosaicism, the mother has no history of neurological disease. Moreover, she did a comprehensive brain MRI protocol which was unremarkable. Clinically, the proband exhibited severe drug-resistant epilepsy with intellectual disability, aligning with more pronounced brain MRI features displaying a thick SBH with fronto-parietal pachygyria. We attribute the proband’s severe phenotype to the nature of the variant, its location in the crucial N-DC, and the presumed de novo transmission.

The c.907C>T (p.Arg303*) variant, identified in an unrelated female proband, was previously reported as pathogenic and was found de novo in a female with anterior predominant pachygyria, hydrocephalus and moderate intellectual disability but without any history of epilepsy [16]. Conversely, our proband has a long-standing drug-resistant epileptic encephalopathy, despite similarities in imaging findings. Our proband’s distinct clinical manifestations underscore the variability in phenotypic expression resulting from the same truncating variant in the *DCX* gene, irrespective of the involvement and distribution of the cortical malformation.

We believe that our report is noteworthy for several reasons. Firstly, we present two novel DCX pathogenic variants, expanding the genotypic–phenotypic correlations of LIS spectrum disorders. Secondly, the cases presented challenge the assumption that the severity of imaging features always corresponds to clinical severity, as exemplified by the unique characteristics of Family 1. Previous studies have shown that the severity of neurological impairment including epilepsy closely correlates with the degree of agyria [18]. While we generally align with this perspective, Family 1 presents an interesting deviation from this conventional understanding. Thirdly, our findings further reinforce general inferences about the greater severity of de novo truncating variants compared to inherited missense ones, as illustrated by the divergent clinical features among the three unrelated probands.

Despite some limitations in our study, such as not testing all known tubulin genes and incomplete parental testing for the proband of Family 3, we believe our documented phenotypic descriptions of the three unrelated families provide valuable insights and stimulate further discussions in DCX-SBH cases.

## 4. Materials and Methods

### 4.1. Cohort Description

Clinical data were collected from probands, their parents, and other relatives. Families were selected from our outpatient epilepsy clinic in Catanzaro, Italy. Three expert epileptologists (A.G., F.F., I.S.) reviewed clinical history of the probands. Pedigrees were constructed extending back as many generations as possible (Figure 2A). All cases were Caucasian and were born in Calabria, in the south of Italy. The fourth-generation Family 1 contains 5 Caucasian individuals, 4 of them affected, all women with a mean age of 44.7 years (range 21–74). Family 2 consists of 3 individuals, while Family 3 contains one affected individual. Comprehensive clinical and laboratory evaluations were obtained from cases and relatives at the time of investigation and from a review of the cases’ medical records. All affected individuals had standard electroencephalogram (EEG) and brain MRI according to the latest (ILAE) recommendations [19].

### 4.2. Next-Generation Sequencing

Informed consent was obtained from each participant. Genomic DNA was extracted from peripheral blood using a Wizard Genomic DNA extraction kit according to the manufacturer’s instructions (Promega, Madison, WI, USA). DNA concentration was evaluated by Qubit^®^ 2.0 Fluorometer^®^ (Thermo Fisher Qubit dsDNA HS Assay Kit, Waltham, MA, USA).

Library preparation was carried out using the Ion Ampliseq library Kit Plus (Life Technologies, CA, USA), starting from 10 ng of gDNA, and the AmpliSeq custom panel that includes the coding regions of 39 genes listed in Supplementary Table S1. Template preparation was performed by the Ion PGM Template OT2 200 kit and Ion OneTouch-2 Instrument. The libraries were further sequenced using Ion PGM-Dx sequencer according to manufacturer’s instructions (Thermo Fisher).

### 4.3. Bioinformatics Analysis

Data analysis was performed using Torrent Suite Software v5.12. The parameters used to filter the variants were: coverage ≥ 100, quality score ≥ 30 and frequency ≥ 5%. The candidate variants were further filtered through the dbSNP141, the 1000 Genomes Project datasets, and 5000Exome database, removing the common variants observed in the general population (MAF > 0.5%).

The resulting variants were annotated according to functional annotation SIFT (http://sift.jcvi.org, accessed on 1 April 2024) and/or Polyphen2 (http://genetics.bwh.harvard.edu/pph2/, accessed on 1 April 2024) algorithms.

### 4.4. Sanger Sequencing

Sanger sequencing was used to validate the variants. All exons and intron–exon boundaries of DCX (NM_001195553.2) were amplified and sequenced using a BigDye Terminator chemistry ver. 3.1 (Life Technologies) on an ABI 3500 Genetic Analyzed (Life Technologies, Carlsbad, CA, USA).

All individuals were also screened for LIS1 and TUBA1A mutations. Segregation analysis was performed on the family members where DNA samples were available.

Multiplex ligation-dependent probe amplification (MLPA) was also performed to exclude genomic rearrangements, using the lissencephaly probe kit (SALSA P61 Lissencephaly, MRC Holland, Amsterdam, The Netherlands).

### 4.5. Predictive Tools

Predictive in silico tools, Mutation Taster (https://www.mutationtaster.org/, accessed on 1 April 2024) and Polyphen-2, were examined to predict the potential pathogenic effect of all identified variants on protein function. The probability of missense variant pathogenicity was predicted using the REVEL Score, CADD-phred Score, MetaDome Score (https://stuart.radboudumc.nl/metadome/, accessed on 1 April 2024) and AlphaMissense [17]. Finally, the variants were classified according to ACMG guidelines [20,21]. We refined the classification based on the PP3/BP4 criteria, incorporating the latest recommendations from ClinGen [22].

## Figures and Tables

**Table 1:** In silico analysis. Exonic variants identified in the DCX gene through targeted panel sequencing analysis in our epilepsy cohort and results of in silico prediction programs.

Family	1	2	3
Variant type	missense	nonsense	nonsense
NT change	c.601A>G	c.210C>G	c.907C>T
AA change	p.Lys201Glu	p.Tyr70*	p.Arg303*
Accession no.	-	rs587783532	rs587783592
gnomAD variant frequency	0	0	0
MutationTaster	disease-causing	disease-causing	disease-causing
PolyPhen2	probably damaging	-	-
Revel	0.916	-	-
CADD-phred	26.20	34.00	36.00
MetaDome	0.25	-	-
Alpha Missense	0.9989	-	-
ACMG classification	likely pathogenic	pathogenic	pathogenic
ACMG criteria	PM1 + PM2 + PP1 + PP3 + PP4	PVS1 + PM2 + PP1	PVS1 + PM2 + PM6 + PP5

NT, nucleotide; AA, amino acid. Significance of the scores: Revel: a score > 0.5 is considered pathogenic. CADD-phred: a score > 20 is considered pathogenic. MetaDome: a score between 0 and 0.5 is considered intolerant, between 0.5 and 0.7 is considered slightly intolerant, and >0.7 is considered neutral. Alpha Missense: a score of 1 is considered pathogenic, 0 is considered benign, and between them is uncertain.

## Data Availability

The data that support the findings of this study are available from the corresponding author (A.G.) upon reasonable request.

## Supplementary Materials

The following supporting information can be downloaded at: https://www.mdpi.com/article/10.3390/ijms25105505/s1.
