# Supplementary material for: Phenotypic Variability in Novel Doublecortin Gene Variants Associated with Subcortical Band Heterotopia
Source: Int J Mol Sci. 2024 May 18;25(10):5505. doi: 10.3390/ijms25105505 (PMC11122195; doi:10.3390/ijms25105505)
Supplement: Supplementary file 1 [file ijms-25-05505-s001.zip › ijms-2993780-supplementary.pdf]

no	Gene
1	ARHGEF9
2	B3GALNT2
3	CDKL5
4	CHD2
5	CHRNA2
6	CHRNA4
7	CHRNB2
8	DCX
9	DEPDC5
10	EPM2A
11	EPM2B
12	FLNA
13	FOXG1
14	GABRA1
15	GABRD
16	GABRG2
17	GLUT1
18	GRIN1
19	HCN1
20	KCNA2
21	KCNQ2
22	KCNQ3
23	KCNT1
24	LGI1
25	NPRL3
26	PCDH19
27	PLCB1
28	PNKP
29	POMGNT1
30	POMGNT2
31	PRRT2
32	RELN
33	SCN1A
34	SCN1B
35	SCN2A
36	SCN8A
37	SCN9A
38	SLC35A2
39	STXBP1

Supplementary Table S1*. *Targeted panel sequencing. *AmpliSeq custom panel, designed to sequence the coding regions of 39 genes, previously associated with lissencephaly and epilepsy.*
